# Dietary intake, energy availability, and power in men collegiate gymnasts

**DOI:** 10.3389/fspor.2024.1448197

**Published:** 2024-09-18

**Authors:** Nicholas M. Kuhlman, Margaret T. Jones, Andrew R. Jagim, Meghan K. Magee, Luke Wilcox, Jennifer B. Fields

**Affiliations:** ^1^ Department of Nutritional Sciences, University of Connecticut, Storrs, CT, United States; ^2^ Patriot Performance Laboratory, Frank Pettrone Center for Sports Performance, George Mason University, Fairfax, VA, United States; ^3^ Sport, Recreation, and Tourism Management, George Mason University, Fairfax, VA, United States; ^4^ Sports Medicine Department, Mayo Clinic Health System, La Crosse, WI, United States; ^5^ Exercise Science and Exercise Physiology, Kent State University, Kent, OH, United States; ^6^ Exercise Science and Athletic Training, Springfield College, Springfield, MA, United States

**Keywords:** low energy availability, body composition, reactive strength index, power output, gymnastics

## Abstract

**Introduction:**

The purpose was to examine the prevalence of low energy availability (LEA), explore dietary behaviors in men collegiate gymnasts (*n* = 14), and investigate the relationships between energy availability (EA), body composition, and plyometric performance.

**Methods:**

Body composition was measured using air displacement plethysmography. Lower- and upper-body peak power (PWRpeak) and modified reactive strength index (RSI_mod_) were calculated from countermovement jump (CMJ) and plyometric push-up (PP) assessments. Energy expenditure was tracked over 3 days, while daily energy and macronutrient intake were recorded. EA was calculated and used to categorize athletes into LEA and non-LEA groups. Pearson correlation coefficients were used to examine relationships between EA, body composition, and performance metrics.

**Results:**

85.7% of athletes (*n* = 12) exhibited LEA (20.98 ± 5.2 kcals/kg FFM), with non-LEA athletes (*n* = 2) marginally surpassing the <30 kcal/kg of fat-free mass (FFM) threshold (30.58 ± 0.2 kcals/kg FFM). The cohort (*n* = 14) consumed insufficient energy (30.5 ± 4.5 kcal/kg/day) and carbohydrates (3.7 ± 1.1 g/kg/day), resulting in LEA (22.36 ± 5.9 kcal/kg/FFM). EA was not correlated with body composition or performance metrics.

**Discussion:**

A high prevalence of LEA may exist in men gymnasts, largely due to a low relative energy and carbohydrate intake.

## 1 Introduction

Gymnastics is a physically demanding sport that requires the combination of strength, muscular endurance, flexibility, coordination, and technical skill to execute intricate routines with precision and control (1). The sport of men's gymnastics includes six events: floor exercise, pommel horse, rings, vault, parallel bars, and high bar (2). Many routines involve a combination of twists, jumps, inversions, and other high-velocity movements resulting in a unique variety of physiological demands and concomitant adaptations (3). Durations of routines vary substantially with event, ranging from approximately 5 s in the vault to 70–90 s on the floor exercise, further highlighting the sport's diverse bioenergetic demands (1). To develop physiological capabilities, gymnasts adhere to rigorous training schedules from a young age, maintaining weekly training loads ranging from 21 to 37 h year-round, which presents long-term challenges to weight management practices (4, 5).

Low body fat to high fat free mass ratios are thought to enhance performance by improving movement efficiency, aesthetic presentation, strength-to-mass ratio, and providing various biomechanical advantages (3, 6). Specifically, leanness is considered a critical body composition characteristic for success, with elite female and male gymnasts maintaining body fat levels ranging from 13%–16% to 8%–12%, respectively (3, 7). Consequently, athletes engaged in aesthetic sports like gymnastics have been found to be at a higher risk of developing behaviors of disordered eating and excessive activity levels in an attempt to minimize fat mass (6). While research on disordered eating in men's gymnastics is limited, reports indicate that nearly 30% of male athletes engaged in sports emphasizing leanness exhibit harmful weight control behaviors (8, 9), marked by restricted energy and carbohydrate intake (10). Additionally, evidence suggests a curvilinear relationship exists between body mass index (BMI) and gymnastics performance, suggesting that excessively low BMI may adversely affect both performance and health (3, 11). Therefore, gymnasts’ pursuit of a lean physique, beyond the point of performance optimization, increases the risk of disordered eating patterns, potentially leading to relative energy deficiencies (5).

Low energy availability (LEA) is a metric used to quantify the residual energy available to support the body's physiological functions after accounting for the energy expenditure from exercise (12–14). LEA is used to identify athletes at risk of Relative Energy Deficiency in Sport (RED-S), which represents a multifactorial physiological dysfunction resulting from chronic energy deficiency, stemming from insufficient energy intake, excessive energy expenditure from training, or a combination of both (15, 16). Calculated energy availability (EA) values for males are: <30 kcal/kg FFM/day, clinical LEA; 30–45 kcal/kg FFM/day, subclinical LEA; ≥40 kcal/kg FFM/day, optimal EA (17). The majority of studies investigating the consequences of LEA have focused predominantly on female athletes (9)**.** Research within women's gymnastics has highlighted the increased risk of LEA among pre-teenage (5), teenage (5), and adult (18, 19) gymnasts. Emerging evidence suggests that male athletes, particularly those in aesthetic-based competitions (18) or weight-category sports (20), are vulnerable to the detrimental effects of LEA (9). Indeed, the 2014 International Olympic Committee's (IOC) consensus statement (21), along with subsequent updates in 2018 (22) and 2023 (23), on RED-S, has broadened the understanding of LEA to include male athletes. While LEA is frequently used to identify risk factors or diagnose RED-S, there is a lack of research investigating the prevalence of LEA among men collegiate gymnasts. Moreover, uncertainties exist regarding the potential impact of LEA on various indices of physical performance, particularly in terms of plyometric performance.

The rapid muscle contractions required during gymnastics routines on apparatuses such as the vault, rings, and floor exercise demand explosive lower- and upper-body power (24). These contractions are driven by the stretch-shortening cycle (SSC), marked by a swift transition between the initial eccentric “stretch” and the subsequent concentric “recoil” (25). The modified reactive strength index (RSI_mod_), which is calculated from jump height, ground contact time, and body mass, serves as a useful indicator of successful technical execution (24), especially for movements requiring muscle re-contraction after landing. While RSI_mod_ focuses on the ability to produce force during the SSC, the measurement of peak power (PWR_peak_) provides distinct yet related information. PWR_peak_ represents the maximum instantaneous power and is crucial for generating the height, rotation, and momentum necessary for movements such as vaulting, tumbling, and dismounting from apparatuses (24). Previous research has examined aspects of upper- and lower-body power in gymnasts including girls (26), women collegiate (27), and boys (28). However, research among men collegiate gymnasts, the metrics of RSI_mod_ and PWR_peak_, and their relationship with EA and body composition remain understudied.

Therefore, the purpose of the current study was to examine the prevalence of LEA and explore dietary behaviors among a convenience sample of National Collegiate Athletic Association (NCAA) Division III collegiate men's gymnastics athletes. A secondary aim was to investigate the relationships between EA, body composition, and upper- and lower-body plyometric performance metrics.

## 2 Materials and methods

### 2.1 Experimental design

This observational study began in August prior to the academic term and gymnastics season. Athletes underwent assessments of body composition, reactive strength index (RSI), and power output during one day of testing. Additionally, a 3-day monitoring period was conducted to evaluate EA. Athletes recorded their dietary intake and were provided with team-based heart rate monitoring systems equipped with inertial sensors to measure activity energy expenditure during practices.

### 2.2 Participants

NCAA Division III men gymnastics athletes (*n* = 14) participated in the current study (age: 21.0 ± 1.2 years; height: 1.66 ± 4.68 m; body mass: 67.6 ± 5.1 kg). Players were medically cleared for intercollegiate athletic participation, had the risks and benefits explained beforehand, signed an institutionally approved written consent form, and completed a medical history form.

### 2.3 Procedures

#### 2.3.1 Body composition

Athletes were instructed to refrain from exercise, eating, and drinking for ≥2 h before testing. Testing was conducted in the early morning after an overnight fast. Upon arrival to the laboratory, height and body mass were recorded to the nearest 0.01 cm and 0.02 kg, respectively, using a stadiometer (Detecto, Webb City, MO, USA) and digital scale (BOD POD model 2000A; BOD POD; Cosmed USA, Concord, CA, USA), with each subject barefoot. Body composition variables (i.e., percent body fat [BF%], fat-free mass [FFM], and fat mass ) were assessed using air displacement plethysmography (BOD POD model 2000A; BOD POD; Cosmed USA, Concord, CA, USA) according to standard operating procedures. Fat-free mass index (FFMI) was calculated using the following equation (29): FFM (kg)/Height^2^ (m^2^).

Before each testing session, calibration procedures were completed according to the manufacturer guidelines using an empty chamber and a calibrating cylinder of a standard volume (49.55 L). Once all tests passed, researchers proceeded with testing. A trained technician performed Bod Pod testing. Participants were instructed to enter the Bod Pod and sit in an erect position with their hands folded in their laps to obtain body volume. Athletes were instructed to wear unpadded compression shorts or spandex and, when applicable, a swim cap with the hair tucked in. They were asked to remove all jewelry to reduce air displacement. Athletes entered the Bod Pod and sat in an upright position with hands folded on their laps. Lung gas volume was estimated using manufacturer guidelines.Two tests were performed to ensure reliability of the assessment. If the tests results were not within 150 ml of each other, 2 more tests were executed to achieve reliable data.

#### 2.3.2 Plyometric performance variables

Lower- and upper-body peak power (PWR_peak_, w) and modified RSI (RSI_mod_, AU) were calculated from countermovement jump (CMJ) and plyometric push-up (PP) assessments, using a commercially available force plate (AccuPower Force Platform, AMTI: Watertown, MA, USA). Athletes were instructed to keep their hands on their hips during the jump and were instructed to maintain straight legs during the flight, before landing on both feet with flexion of the hips, knees, and ankles. For the PP, athletes were positioned with their hands right below their shoulders and their feet shoulder-width apart. When instructed, athletes lowered their elbows to the height of their shoulders and pushed up as fast and as forcefully as they could. A trial was discarded and repeated when a participant did not lower their elbows to their shoulder height as visually inspected by the same trained researchers. Three attempts of CMJ and PP were conducted per assessment, with a minimum of 2-min rest between each trials to minimize fatigue effects.

PWR_peak_ was automatically yielded from AMTI's data acquisition software (NetForce, Watertown, MA, USA). RSI_mod_ was subsequently computed by normalizing force plate-derived jump height and ground contact time with respect to each athlete's body mass:

$$\mathrm{RS}I_{\mathrm{mod}}=\frac{\mathrm{Jump}\;\mathrm{Height}}{\mathrm{Ground}\;\mathrm{Contact}\;\mathrm{Time}\;X\;\sqrt{\mathrm{Body}}\mathrm{Mass}}$$

#### 2.3.3 Energy availability

Athletes were asked to record dietary intake using an online commercially available nutrition analysis program (MyFitnessPal, Under Armour, Baltimore, MD, USA). Prior to this period, participants attended an informational session where they were educated on methods to estimate portion sizes accurately. This session included detailed descriptions and demonstrations of portion size estimatation techniques. Additionally, they were provided with informational packets and instructional videos to reinforce accurate self-reporting. Daily energy and macronutrient intakes were averaged across the 3-day monitoring period. Absolute energy and macronutrient intakes (kcal/day or g/day) were recorded and were expressed relative to body weight (kcal/kg/day or g/kg/day) to enable comparison between individuals. During the same 3-day period, activity energy expenditure was monitored via wearable monitoring devices (Polar H9, Polar Electro, Kempele, Finland) and calculated from proprietary algorithms. These devices have been used in previous studies to measure activity energy expenditure in athletic populations (30). EA was then calculated by subtracting the activity energy expenditure from energy intake and expressed as kcals per kilogram of FFM. A threshold of <30 kcal/kg of FFM was used to classify athletes as having LEA.

### 2.4 Statistical analysis

SPSS version 29.0 (IBM Corp., Armonk, NY, USA) was used for summary statistics. Athletes were categorized into LEA vs. non-LEA groups to observe differences in body composition, EA, and plyometric performance variables. Absolute and relative carbohydrate, protein, and fat intake were also analyzed. However, due to the small sample size of athletes in the non-LEA group (*n* = 2), inferential statistics were de-emphasized and a descriptive analysis (mean ± SD, range) was prioritized. Pearson correlation coefficients were used to examine relationships between EA, body mass, fat-free mass, fat mass, body fat percentage, CMJ-height, CMJ-RSI_mod_, CMJ-PWR_peak_, PP-height, PP-RSI_mod_, and PP-PWR_peak_. The following criteria were used for interpreting the correlation coefficients: very weak: <0.20; weak: 0.20–0.39; moderate: 0.40–0.59; strong: 0.60–0.79; and very strong: >0.80 (*p* < 0.05) (31).

## 3 Results

A summary of anthropometric, body composition, and demographic data stratified by energy status are presented as mean ± SD in Table 1. All athletes exhibited body fat levels below 10% (9.2 ± 3.5%; range: 5.7–19.1), with a body mass of 67.6 ± 5.1 kg (range: 57.5–76.0), FFM of 61.3 ± 5.7 kg (range: 52.1–69.9), FFMI of 22.12 ± 1.7 kg m^2^ (range: 18.93–24.76), and FM of 6.15 ± 2.2 kg (range: 3.5–12.3).

**Table 1:** Summary of subject anthropometric, body composition and demographic data, stratified by energy status (*n* = 14).

	LEA (*n* = 12)	Non-LEA (*n* = 2)	All (*n* = 14)
Height (m)	1.66 ± 4.66	1.66 ± 6.8	1.66 ± 4.68
Body mass (kg)	67.9 ± 5.4	64.4 ± 2.2	67.6 ± 5.1 [1.36]
Fat-free mass (kg)	61.7 ± 6.1	58.7 ± 0.6	61.3 ± 5.7 [1.52]
FFMI (kg·m^2^)	22.78 ± 1.7	21.29 ± 1.9	22.12 ± 1.7
Fat mass (kg)	6.23 ± 2.37	5.74 ± 1.59	6.15 ± 2.2 [0.59]
Body fat (%)	9.2 ± 3.8	8.9 ± 2.2	9.2 ± 3.5 [0.94]
Age (years)	21.1 ± 1.3	20.5 ± 0.7	21.0 ± 1.2

Values are mean ± standard deviation; LEA, low energy availability; FFMI, fat-free mass index. Values within brackets represent the standard error of the estimate (SEE) for BodPod variables.

A summary of dietary intake (energy, carbohydrates, protein, fat), activity energy expenditure, and EA, stratified by LEA status is provided in Table 2. A total of 85.7% of athletes (*n* = 12) exhibited LEA (20.98 ± 5.2 kcals/kg FFM), with non-LEA athletes (*n* = 2) marginally surpassing the <30 kcal/kg of FFM threshold (30.58 ± 0.2 kcals/kg FFM), thus qualifying for subclinical LEA (30–40 kcal/kg/FFM) (9, 17). The entire cohort (*n* = 14) consumed insufficient energy (30.5 ± 4.5 kcal/kg/day; range: 23.5–39.0), resulting in LEA (22.36 ± 5.9 kcal/kg/FFM; range: 12.0–30.7). Carbohydrate intake was low (3.7 ± 1.1 g/kg/day; range: 2.6–7.0), likely contributing to low energy intake. Relative protein and fat intakes for the cohort were 1.6 ± 0.4 kg (range: 90.3–113.6) and 0.9 ± 0.3 kg (range: 53.0–88.3), respectively. Average activity energy expenditure for the 3-day monitoring period was 685.8 ± 170.4 kcal (range: 400.0–1,003.0).

**Table 2:** A summary of average daily activity energy expenditure and macronutrient intake by energy status.

		LEA (*n* = 12)	Non-LEA (*n* = 2)	All (*n* = 14)
Intake	Energy Intake (kcal/day)	1,987.4 ± 268.3	2,051.8 ± 300.8	2,051.8 ± 300.8
	Relative energy intake (kcal/kg/day)	29.3 ± 3.5	37.8 ± 1.7	30.5 ± 4.5
	Carbohydrate intake (g/day)	246.8 ± 81.9	278.0 ± 96.2	251.2 ± 80.7
	Relative carbohydrate intake (g/kg/day)	3.6 ± 1.1	4.3 ± 1.3	3.7 ± 1.1
	Protein intake (g/day)	110.6 ± 32.8	102.0 ± 16.5	109.4 ± 230.7
	Relative protein intake (g/kg/day)	1.6 ± 0.4	1.6 ± 0.4	1.6 ± 0.4
	Fat intake (g/day)	66.5 ± 16.6	70.7 ± 24.9	67.1 ± 16.8
	Relative fate intake (g/kg/day)	0.98 ± 0.2	1.1 ± 0.4	0.9 ± 0.3
Expenditure	Activity energy expenditure (kcal)	692.9 ± 177.8	654.5 ± 159.1	685.8 ± 170.4
status	Energy availability (kcals/kg FFM/day)	20.98 ± 5.2	30.58 ± 0.2	22.36 ± 5.9

Values represented as mean ± standard deviation; LEA, low energy availability; FFM, fat-free mass.

A summary of average movement height, RSI_mod_, and PWR_peak_ for CMJ and PP, stratified by LEA status is included in Table 3. For lower body, athletes had a CMJ-height of 0.38 ± 0.05 m (range: 0.29–0.46), RSI_mod_ of 0.48 ± 0.10 AU/kg (range: 0.31–0.64), and a PWR_peak_ of 3,663.9 ± 563.6 w (range: 2,362.6–4,662.6). For upper body, athletes had a PP-height of 0.25 ± 0.09 m (range: 0.12–0.42), RSI_mod_ of 0.19 ± 0.06 AU/kg (range: 0.11–0.29), and PWR_peak_ of 1,678.9 ± 295.3 w (range: 1,233.8–2,301.9).

**Table 3:** A summary of average upper and lower body plyometric performance variables.

		LEA (*n* = 12)	Non-LEA (*n* = 2)	All (*n* = 14)
CMJ	Height (m)	0.37 ± 0.05	0.39 ± 0.09	0.38 ± 0.05
	RSI_mod_ (AU)	0.47 ± 0.09	0.54 ± 0.14	0.48 ± 0.10
	PWR_peak_ (w)	3,656.4 ± 612.3	3,708.6 ± 36.4	3,663.9 ± 563.6
PP	Height (m)	0.24 ± 0.09	0.31 ± 3.4	0.25 ± 0.09
	RSI_mod_ (AU)	0.19 ± 0.06	0.22 ± 0.09	0.19 ± 0.06
	PWR_peak_ (w)	1,644.6 ± 303.9	1,885.1 ± 133.7	1,678.9 ± 295.3

Values represented as mean ± standard deviation; CMJ, countermovement jump; PP, plyo push-up; RSI_mod_, modified reactive strength index; PWR_peak_, peak power; LEA, low energy availability.

Relationships between EA, body composition metrics, and upper- and lower-body plyometric metrics are presented in Table 4. EA was not correlated with body composition metrics nor upper- or lower-body performance metrics. BF% was correlated with FFM (*p* = 0.025; 95% CI: −0.855, 0.093), FM [*p* < 0.001; 95% CI: (0.95, 0.995)], CMJ-height [*p* = 0.012; 95% CI: (−0.877, −0.177)], and CMJ-RSI_mod_ [*p *= 0.43; 95% CI: (−0.835, −0.024)]. FM was correlated with CMJ-height [*p* = 0.008; 95% CI: (−0.888, −0.226)] and CMJ-RSI_mod_ [*p* = 0.037; 95% CI: (−0.841, −0.043)]. CMJ-height was correlated with CMJ-RSI_mod_ [*p* < 0.01; 95% CI: (0.401, 0.923)] and CMJ-PWR_peak_ [*p* = 0.04; 95% CI: (0.031, 0.838)]. CMJ-RSI_mod_ was corelated with CMJ-PWR_peak_ [*p* = 0.025; 95% CI: (0.091, 0.855)]; PP-height was correlated with PP-PWR_peak_ [*p* < 0.001; 95% CI: (0.537, 0.945)].

**Table 4:** Relationships between energy availability, body composition, and upper- and lower-body plyometric variables.

	Body mass (kg)	BF% (%)	FFM (kg)	FFMI (kg·m^2^)	FM (kg)	EA (kcal/kg FFM)	REI (kcal)	CMJ height (m)	CMJ RSI_mod_ (AU)	CMJ PWR_peak_ (w)	PP Height (m)	PP RSI_mod_ (AU)	PP PWR_peak_ (w)
Body mass (kg)	1	−0.234	0.921^b^	0.732^b^	−0.063	−0.101	−0.227	−0.103	−0.013	−0.264	0.0,264	0.324	0.096
BF% (%)		1	−0.595^a^	−0.504	0.984^b^	0.136	−0.082	−0.647^a^	−0.547^a^	−0.445	−0.219	−0.051	−0.102
FFM (kg)			1	0.806^b^	−0.447	−0.140	−0.156	0.172	0.207	0.442	−0.138	0.285	0.116
FFMI (kg·m^2^)				1	−0.385	−0.301	−0.261	0.082	0.110	0.210	−0.108	0.493	−0.009
FM (kg)					1	0.125	−0.122	−0.675^b^	−0.561^a^	−0.391	−0.252	0.014	−0.076
EA (kcal/kg FFM)						1	0.845^b^	0.212	0.206	0.289	0.339	0.187	0.408
REI (kcal)							1	0.273	0.136	0.254	0.474	0.146	0.520
CMJ-Height (m)								1	0.768^b^	0.553^a^	0.324	0.103	0.144
CMJ-RSI_mod_ (AU)									1	0.593^a^	0.088	−0.015	−0.059
CMJ-PWR_peak_ (w)										1	0.458	0.051	0.483
PP-Height (m)											1	0.039	0.831^b^
PP-RSI_mod_ (AU)												1	0.0–82
PP-PWR_peak_ (w)													1

^a^ Correlation is significant at the 0.05 level; ^b^Correlation is significant at the 0.01 level; BF%, percent body fat; FFM, fat-free mass; FM, fat mass; REI, relative energy intake; EA, energy availability; CMJ, countermovement jump; PP, plyometric push-up; RSI_mod_, modified reactive strength index; PWR_peak_, peak power.

## 4 Discussion

The primary aim of the current study was to examine the prevalence of LEA and to explore dietary behaviors among a convenience sample of men collegiate gymnasts. Of note, 85.7% of the athletes within the cohort experienced LEA, as determined through direct assessment of dietary intake and activity energy expenditure. Additionally, the cohort consumed inadequate energy (30.5 ± 4.5 kcal/kg/day), largely influenced by a low carbohydrate intake (3.7 ± 1.1 g/kg/day), leading to the observed state of LEA (22.36 ± 5.9 kcals/kg FFM/day). No significant correlation existed between EA and either body composition or upper- and lower-body plyometric performance metrics. This study is novel as it marks the first instance of LEA prevalence reported in a sample of male gymnasts.

Previous reports in women's gymnastics indicate LEA prevalence rates range from 35%–56% for adult recreational and elite athletes (18), and 75% for teenage athletes (5), which are all lower than the prevalance of LEA observed in the current study. Moreover, the high prevalence of LEA observed in men gymnasts surpasses previous findings in men's dancing (29%) (32), a similar aesthetic sport emphasizing weight-control behavior. However, it is important to consider that LEA assessment in the study of men's dancing was conducted using the Dance-Specific Energy Availability Questionnaire (DEAQ), underscoring the challenge of making direct comparisons between studies due to differences in measurement techniques (9). LEA prevalence rates of 25% (33), 30% (34), 66% (35), and 70% (10), have been reported in male endurance athetes from non-aesthetic sports like cycling, distance running, and race walking. While LEA rates are notably lower than those observed in the current study, these studies had larger sample sizes (*n* = 21–53) and longer assessment durations (≥7 days). The absence of a standardized reference time frame for dietary or exercise assessments complicates absolute comparisons of LEA prevalence (36). Further, these studies involved well-trained, elite adult, or masters participants (age range: 18–50 years), potentially lacking the unique constraints experienced by the collegiate athletes in the current study, such as limited fueling opportunites due to demanding academic schedules, which could hinder access to optimal nutritional resources (37). It is important to note that the cross-sectional nature of these studies makes it unclear whether the causes of LEA are intentional, such as disordered eating behaviors, or inadvertent, stemming from factors like high-volume training or lack of education (9, 21, 38). With the exception of high-level endurance sports, which require high-volumes of training, low energy intake likely underpins the high prevalence of LEA for most aesthetic sport athletes. For example, the activity energy expenditure observed in the current study (692.9 ± 177.8 kcal) would not be considered an excessively high activity level. As such, it is apparent that nutrient deficiencies, primarily insufficient macronutrient energy consumption, plays a major role in influencing LEA prevalence (10).

The mean EA value observed in the current study was 22.36 ± 5.9 kcals/kg FFM/day, falling below the threshold of <30 kcals/kg FFM/day used to classify individuals with LEA (17). Surprisingly, it is lower than previously reported values in pre-teenage (60.04 kcal/kg FFM/day), teenage (29.7 kcal/kg FFM/day), and adult (31.5 kcals/kg FFM/day) female gymnasts (5, 19). Differences in sample size, measurement techniques, and assessment durations may partially explain the disparities in EA. However, the notably lower EA observed in the current study suggests potential issues with energy intake among men collegiate gymnasts that warrant further investigation. In part, lower EA values may be explained by the greater amounts of FFM in male gymnasts compared to females. It is plausible that men and women gymnasts may have comparable levels of energy expenditure; thus if the men neglected to compensate by increasing energy intake, lower EA values will result. The mean EA for athletes in the current study is comparable to previous season average values reported for male cyclists (20 kcals/kg FFM/day) (10), but appears lower than previously reported preseason values for male distance runners and racewalkers (27–36 kcals/kg FFM/day) (39, 40). Further, the mean FFM (61.3 ± 5.7 kg) for the gymnasts is comparable to that previously reported for male cyclists (63.8 ± 3.8 kg). These findings contribute to the growing body of evidence suggesting that male athletes are vulnerable to the adverse effects of LEA, particularly those engaged in high-volume training (e.g., distance runners), weight-category sports (e.g., wrestlers), and aesthetic-based competitions (e.g., gymnasts, dancers) (9) due to their higher energy requirements and greater amounts of FFM.

The primary contributor of LEA appears to stem from insufficient energy intake, namely from a low carbohydrate intake. Scientific societies have provided recommendations regarding the optimal carbohydrate intake for athletes based on their specific sports disciplines (41, 42). For men's and women's gymnastics, a relative carbohydrate intake of 5–7 g/kg/day is suggested (42). However, while previous reports indicated an average carbohydrate intake of 5.1 ± 2.3 g/kg/day for adult female gymnasts without LEA (19), gymnasts in the current study consumed 3.7 ± 1.1 g/kg/day, a level similar to that reported for teenage female gymnasts with LEA (3.7 ± 1.3 g/kg/day) (5). Given the limited evidence indicating substantial differences in carbohydrate utilization between adolescents and adults (43), it seems that inadequate carbohydrate intake is the most probable contributor to energy deficiency in male and female gymnastics athletes, irrespective of age (5, 43). In support, protein (1.6 ± 0.4 kcal/kg/day) and fat (0.9 ± 0.3 kcal/kg/day) intake were within appropriate ranges (41, 42) considering the type and amount of physical activity performed by the men gymnasts. However, the focus on leanness in gymnastics often results in weight-control measures, including carbohydrate restriction, a trend also noted in competitive male cyclists (10). Research highlights the prevalence of fad diets advocating carbohydrate restriction in sports emphasizing leanness (10), which stresses the importance of sport nutrition education, practical dietary skills development, and behavior change interventions, which may be necessary to effectively address nutritional deficiencies among athletes (16).

The current investigation is the first to report PWR_peak_ and RSI_mod_ values for a sample of collegiate men gymnasts. Notably, the CMJ-PWR_peak_ (3,663.9 ± 563.6 w) exceeds previous reports for NCAA DI women gymnasts (32,100 ± 350 w) (27) and substantially surpasses findings for male youth gymnasts (1,400 ± 543 w) (28). While average RSI_mod_ has not been documented in other gymnastics populations (e.g., women, youth), the observed average RSI_mod_ in current study (0.48 ± 0.10 w) surpasses values reported for men's baseball (0.41 ± 0.08 w), men's tennis (0.30 ± 0.07 w), and men's soccer (0.44 ± 0.05 w) athletes (44). The notably higher RSI_mod_ observed in men gymnasts compared to athletes in field or court sports underscores the unique demands of gymnastics, where athletes heavily rely on the SSC during plyometric activities, as they maneuver their bodies acrobatically on various apparatuses. Over time, this likely leads to sport-specific adaptations, which help develop lower-body power and explosiveness. Currently, PP-PWR_peak_ (1,678.9 ± 295.3 w) or PP-RSI_mod_ (0.19 ± 0.06 w) values have not been reported for gymnastics or other aesthetic sport athletes.

A secondary aim of the current study was to investigate the relationships between EA, body composition metrics, and upper- and lower-body plyometric performance metrics, namely RSI_mod_ and PWR_peak_, in a sample of collegiate men's gymnastics athletes. While no correlations were found, this finding contrasts with a recent study conducted by Jurov et al., which observed decreases in CMJ height following 14 days of induced LEA with a 50% reduction in EA in trained male endurance athletes (45). In a subsequent study, the same research group observed decreases in PWR_peak_, relative power, and CMJ-height following 14 days of induced LEA with a 25% reduction in EA (46). In a final study, where EA was reduced in progressive stages of 25%, 50%, and 75%, Jurov et al. discovered CMJ height decreased proportionately with increased EA reductions, and most of the negative effects occurred in the range of 9–25 kcals/kg FFM/day (47). Differences between the aforementioned studies and the current one in subject populations, sport demands, and study design, may account for the disparate results between EA and plyometric performance. However, it is noteworthy that the EA observed in the current study (22.36 ± 5.9 kcals/kg FFM/day) falls within the upper range associated with the most pronounced negative effects on performance as reported by Jurov et al. (47). Further, reports suggesting that suboptimal EA detrimentally affects explosive power even prior to hormonal changes indicate the potential utility of monitoring changes in plyometric performance as early indications of the adverse effects of LEA (46). However, more research is needed to elucidate the specific impact of LEA on plyometric performance in men's gymnastics. Additionally, the current study reported significant negative associations (*r* = −0.547) between CMJ-RSI_mod_ and BF%, emphasizing the importance of maintaining low BF%. This is critical for not only enhancing movement efficiency and promoting aesthetic presentation but also for optimizing strength-to-mass ratio and gaining various biomechanical advantages during set routines (3). Lastly, our findings revealed no significant correlation between EA and body composition variables, contrasting with reports from studies on athletes in other sports (48). This may be due to the lack of standardized protocols for calculating EA, potential errors in self-reported dietary intake and activity expenditure measurements, and individual variability in physiological responses (49). The complex nature of body composition changes in response to EA further complicates the detection of clear correlations (49). Future research with larger sample sizes, accurate measurement techniques, and standardized protocols is necessary to elucidate this relationship.

While the current study has provided valuable insights into dietary behaviors and plyometric performance for a sample of collegiate male gymnasts, a few limitations should be considered. First, the study's small sample size (*n* = 14) may limit the generalizability of the findings. Additionally, the reliance on a single time point, heart rate-derived activity energy expenditure measures, and self-reported dietary intake might not fully capture the dynamic nature of athletes’ energy balance. It should also be noted that the Polar H9 heart rate monitors, used in this study to estimate activity energy expenditure, have not been validated for measuring exercise energy expenditure in activities like gymnastics that often involve substantial anaerobic components. Further, under-reporting is a common issue in dietary self-reporting, and there is the potential for subjects to alter their dietary intake during the recording period, which might not reflect their long-term intake patterns (49). Lastly, the lack of information on whether certain athletes were undergoing intentional weight-cutting during data collection is a notable limitation, as such phases could influence energy intake and expenditure, potentially skewing the results. Future research with larger cohorts and longitudinal designs is warranted to clarify these relationships in greater detail.

## 5 Conclusion

The current research is novel in that it provides the first report of LEA prevalence and upper- and lower-body plyometric performance metrics in a sample of men collegiate gymnasts. Results indicate there may be a high prevalence of LEA, underscoring the need for attention to nutritional practices within this population. Findings suggest that athletes are underfueling, particularly in terms of carbohydrates, which is critical for meeting the energy demands of intense gymnastics training and competition. Failure to address LEA may have implications for performance outcomes among athletes participating in weight-control sports. While the relationship between EA and plyometric performance has been documented in sports such as distance running, it has yet to be determined in men's gymnastics, underscoring the need for further research. Additionally, prolonged LEA can compromise various aspects of athlete health, including bone metabolism, hormonal function, and immune response, thereby elevating the risk of injuries and illness. Interventions aimed at optimizing EA and carbohydrate intake are important for safeguarding the well-being, and potentially physical performance, of men collegiate gymnasts.

## Data Availability

The raw data supporting the conclusions of this article will be made available by the authors, without undue reservation.
